# Decibel Hell: The Effects of Living in a Noisy World

**DOI:** 10.1289/ehp.113-a34

**Published:** 2005-01

**Authors:** Ron Chepesiuk

It’s not difficult for a person to encounter sound at levels that can cause adverse health effects. During a single day, people living in a typical urban environment can experience a wide range of sounds in many locations, including shopping malls, schools, the workplace, recreational centers, and the home. Even once-quiet locales have become polluted with noise. In fact, it’s difficult today to escape sound completely. In its 1999 *Guidelines for Community Noise*, the World Health Organization (WHO) declared, “Worldwide, noise-induced hearing impairment is the most prevalent irreversible occupational hazard, and it is estimated that 120 million people worldwide have disabling hearing difficulties.” Growing evidence also points to many other health effects of too much volume.

The growing noise pollution problem has many different causes. Booming population growth and the loss of rural land to urban sprawl both play a role. Other causes include the lack of adequate anti-noise regulations in many parts of the world; the electronic nature of our age, which encourages many noisy gadgets; the rising number of vehicles on the roads; and busier airports. The U.S. Environmental Protection Agency (EPA) has long identified transportation—passenger vehicles, trains, buses, motorcycles, medium and heavy trucks, and aircraft—as one of the most pervasive outdoor noise sources, estimating in its 1981 *Noise Effects Handbook* that more than 100 million people in the United States are exposed to noise sources from traffic near their homes.

Some experts define noise simply as “unwanted sound,” but what can be unwanted for one person can be pleasant or even essential sound to to another—consider boom boxes, car stereos, drag races, and lawn mowers in this context. Sound intensity is measured in decibels (dB); the unit A-weighted dB (dBA) is used to indicate how humans hear a given sound. Zero dBA is considered the point at which a person begins to hear sound. A soft whisper at 3 feet equals 30 dBA, a busy freeway at 50 feet is around 80 dBA, and a chain saw can reach 110 dBA or more at operating distance. Brief exposure to sound levels exceeding 120 dBA without hearing protection may even cause physical pain.

Mark Stephenson, a Cincinnati, Ohio–based senior research audiologist at the National Institute for Occupational Safety and Health (NIOSH), says his agency’s definition of hazardous noise is sound that exceeds the time-weighted average of 85 dBA, meaning the average noise exposure measured over a typical eight-hour work day. Other measures and definitions are used for other purposes. For example, “sound exposure level” accounts for variations in sound from moment to moment, while “equivalent sound level” determines the value of a steady sound with the same dBA sound energy as that contained in a time-varying sound.

## Growing Volume

In the United States, about 30 million workers are exposed to hazardous sound levels on the job, according to NIOSH. Industries having a high number of workers exposed to loud sounds include construction, agriculture, mining, manufacturing, utilities, transportation, and the military.

Noise in U.S. industry is an extremely difficult problem to monitor, acknowledges Craig Moulton, a senior industrial hygienist for the Occupational Safety and Health Administration (OSHA). “Still,” he says, “OSHA does require that any employer with workers overexposed to noise provide protection for those employees against the harmful effects of noise. Additionally, employers must implement a continuing, effective hearing conservation program as outlined in OSHA’s Noise Standard.”

Meanwhile, there is no evidence to suggest things have gotten any quieter for residents since the EPA published its 1981 handbook. “For many people in the United States, noise has drastically affected the quality of their lives,” says Arline L. Bronzaft, chair of the Noise Committee of the New York City Council of the Environment and a psychologist who has done pioneering research on the effects of noise on children’s reading ability. “My daughter lives near La Guardia airport in New York City, and she can’t open a window or enjoy her backyard in the summer because of the airplane noise.”

Indeed, the term *secondhand noise* is increasingly used to describe noise that is experienced by people who did not produce it. Anti-noise activists say its effect on people is similar to that of secondhand smoke. “Secondhand noise is really a civil rights issue,” says Les Blomberg, executive director of the Noise Pollution Clearinghouse, an anti-noise advocacy group based in Montpelier, Vermont. “Like secondhand smoke, it’s put into the environment without people’s consent and then has effects on them that they don’t have any control over.”

Secondhand noise can also have a negative effect in the workplace. “Workers in the construction trades get exposure to noise not just from what they are doing but also from what is going on around them,” says Rick Neitzel, director of communications for the National Hearing Conservation Association. “Electricians, for example, have a reputation as being a member of a quiet trade, but if they work all day next to a laborer who is using a jackhammer, it’s going to have a harmful effect.”

Even disregarding other people’s noise, there are any number of household tools and appliances that can produce harmful sound levels in the comfort of one’s own home. According to the fact sheet “Noise in the Home” produced by the League for the Hard of Hearing, dishwashers, vacuum cleaners, and hair dryers can all reach or exceed 90 dBA.

Our modern industrialized society has spawned ubiquitous entertainment and sports industries with their boom boxes, “personal stereos” (Gap Kids now even offers a jacket with a built-in radio and speakers conveniently attached right in the hood), surround-sound movie theaters, loud TV commercials, and even louder commercials at sports stadiums crammed full of thousands of noisy fans. In drag racing, a growing international sport, a German team of audio engineers set an earsplitting record of 177 dB–sound pressure level in 2002. Popular “boom cars” equipped with powerful stereo systems that are usually played with the volume and bass turned up abnormally high and the car windows rolled down can hit 140–150 dBA. Listening to music at a level of 150 dBA would be like standing next to a Boeing 747 airplane with its engines at full throttle, according to statistics provided by Noise Free America, an anti-noise advocacy group.

Even the countryside is not immune to the impact of noise pollution. According to the New York Center for Agricultural Medicine and Health in Cooperstown, a staggering 75% of farmworkers have some kind of hearing problem, largely the result of long-term exposure to loud equipment.

The United States is not the only country where noise pollution is affecting the quality of life. In Japan, for instance, noise pollution caused by public loudspeaker messages and other forms of city noise have forced many Tokyo citizens to wear earplugs as they go about their daily lives. In Europe, about 65% of the population is exposed to ambient sound at levels above 55 dBA, while about 17% is exposed to levels above 65 dBA, according to the European Environment Agency.

“The noisy problems associated with air travel are concentrated in communities around airports, whereas motorways or high-speed trains—traveling, for instance, from north to south Europe—have the potential to disturb thousands of people living along the route day after day,” says Ken Hume, a principal lecturer in human physiology at the Manchester Metropolitan University in England.

Noise is indeed everywhere, and experts expect no decrease in noise levels, given the powerful impact of technology on modern life. “In the past three decades, we have built noisier and noisier devices that are not subject to any regulations,” Blomberg says. “Think about it. The car alarm is a seventies invention, as is the leaf blower. The stereo sound systems we have in our cars are much louder than the sound system the Beatles used for their concerts in the sixties. All they had back then were three-hundred-amp speakers.”

## Scary Sound Effects

Numerous scientific studies over the years have confirmed that exposure to certain levels of sound can damage hearing. Prolonged exposure can actually change the structure of the hair cells in the inner ear, resulting in hearing loss. It can also cause tinnitus, a ringing, roaring, buzzing, or clicking in the ears. The American Tinnitus Association estimates that 12 million Americans suffer from this condition, with at least 1 million experiencing it to the extent that it interferes with their daily activities.

NIOSH studies from the mid to late 1990s show that 90% of coal miners have hearing impairment by age 52—compared to 9% of the general population—and 70% of male metal/nonmetal miners will experience hearing impairment by age 60 (Stephenson notes that from adolescence onward, females tend to have better hearing than males). Neitzel says nearly half of all construction workers have some degree of hearing loss. “NIOSH research also reveals that by age twenty-five, the average carpenter’s hearing is equivalent to an otherwise healthy fifty-year-old male who hasn’t been exposed to noise,” he says.

“Noise has an insidious effect in that the more exposure a person has to noise, the more the hearing loss will continue to grow,” says Josara Wallber, disabilities services liaison for the National Technical Institute for the Deaf in Rochester, New York. “Hearing loss is irreversible. Once hearing is lost, it’s lost forever.”

William Luxford, medical director of the House Ear Clinic of St. Vincent Medical Center in Los Angeles, points out one piece of good news: “It’s true that continuous noise exposure will lead to the continuation of hearing loss, but as soon as the exposure is stopped, the hearing loss stops. So a change in environment can improve a person’s hearing health.”

For many young people, changing their environment and their behavior would be a wise and healthy move. That’s because audiologists are fitting more and more of them with hearing aids, says Rachel Cruz, a research associate at the House Ear Clinic. She says audiologists are blaming this disturbing development on youth’s penchant for listening to loud music, especially with the use of headphones.

Research is catching up with this anecdotal evidence. In the July 2001 issue of *Pediatrics*, researchers from the Centers for Disease Control and Prevention reported that, based on audiometric testing of 5,249 children as part of the Third National Health and Nutrition Examination Survey, an estimated 12.5% of American children have noise-induced hearing threshold shifts—or dulled hearing—in one or both ears. Most children with noise-induced hearing threshold shifts have only limited hearing damage, but continued exposure to excessive noise can lead to difficulties with high-frequency sound discrimination. The report listed stereos, music concerts, toys (such as toy telephones and certain rattles), lawn mowers, and fireworks as producing potentially harmful sounds.

For the baby boom generation, on the other hand, a change of environment may be too late. “Many baby boomers began losing their hearing when the amplification of popular music came into vogue in the nineteen sixties,” says Cruz. “We are starting to see that a lot of musicians and audio engineers who have been involved with popular music for a long time are having hearing problems.” Cruz is gathering data for a research study to examine how these professionals’ occupational sound exposures affect their hearing over a span of years.

## Beyond the Ears

The effects of sound don’t stop with the ears. Nonauditory effects of noise exposure are those effects that don’t cause hearing loss but still can be measured, such as elevated blood pressure, loss of sleep, increased heart rate, cardiovascular constriction, labored breathing, and changes in brain chemistry. According to the WHO *Guidelines for Community Noise*, “these health effects, in turn, can lead to social handicap, reduced productivity, decreased performance in learning, absenteeism in the workplace and school, increased drug use, and accidents.”

The nonauditory effects of noise were noted as early as 1930 in a study published by E.L. Smith and D.L. Laird in volume 2 of the *Journal of the Acoustical Society of America*. The results showed that exposure to noise caused stomach contractions in healthy human beings. Reports on noise’s nonauditory effects published since that pioneering study have been both contradictory and controversial in some areas.

Data pertaining to whether noise can increase the risk of damage to the fetus is a case in point. A study published by L.D. Edmonds, P.M. Layde, and J.D. Erickson in the July–August 1979 issue of the *Archives of Environmental Health* found no significant data suggesting an effect of noise on fetal development in pregnant women who lived near airports. But in the October 1997 issue of *Pediatrics*, the Committee on Environmental Health of the American Academy of Pediatrics published a policy statement based on a review of research on the potential health effects of noise on the fetus and the newborn. The committee concluded that excessive noise exposure *in utero* may result in high-frequency hearing loss in newborns and further that excessive sound levels in neonatal intensive care units may disrupt the natural growth and development of premature infants. It recommended that noise-induced health effects on fetuses and newborns are clinical and public health concerns that merit further study.

Studies have revealed that as children grow they are exposed to sounds that can threaten their health and cause learning problems. For instance, in the September 1997 issue of *Environment and Behavior*, Cornell University environmental psychologists Gary Evans and Lorraine Maxwell reported that the constant roar of jet aircraft could cause higher blood pressure, boosted stress levels, and other effects with potential life-long ramifications among children living in areas under the flight paths of airport.

Other human and animal studies also have linked noise exposure to chronic changes in blood pressure and heart rate. For example, in the July–August 2002 issue of the *Archives of Environmental Health,* a team of government and university researchers concluded that exposure to sound “acts as a stressor—activating physiological mechanisms that over time can produce adverse health effects. Although all the effects and mechanisms are not elucidated, noise may elevate systolic blood pressure, diastolic blood pressure, and heart rate, thus producing both acute and chronic health effects.”

Noise has also been shown to affect learning ability. In 1975 Bronzaft collaborated on a study of children in a school near an elevated train track that showed how exposure to noise can affect children’s reading ability. Half of the students in the study were in classrooms facing the train track and the other half were in classrooms in the school’s quieter back section. The findings, published in the December 1975 issue of *Environment and Behavior*, were that students on the quieter side performed better on reading tests, and by sixth grade they were a full grade point ahead of the students in the noisier classrooms.

Bronzaft and the school principal persuaded the school board to have acoustical tile installed in the classrooms adjacent to the tracks. The Transit Authority also treated the tracks near the school to make them less noisy. A follow-up study published in the September 1981 issue of the *Journal of Environmental Psychology* found that children’s reading scores improved after these interventions were put in place. “After we did the study, more than twenty-five other studies were done examining the effect of noise on children’s learning ability,” Bronzaft says. “They have all found the same thing to be true: noise can affect children’s learning.”

The EPA reported in the *Noise Effects Handbook* that surveys taken in communities significantly affected by noise indicated that interruption of sleep was the underlying cause of many people’s complaints. Research has shown that unwanted sound is most annoying at the times when people expect to rest or sleep, that it can interrupt or delay sleep, and that it can have subtle effects on sleep, such as causing shifts from deeper to lighter sleep stages. “The research is pretty solid that noise can prevent people from getting a good night’s sleep,” Hume says. “I believe that sleep deprivation can have negative health effects when it becomes a chronic problem.”

## Fighting for Quiet

Worldwide, airports have become a flash point for community frustration over noise pollution. In September 2002, officials at the Frankfurt am Main Airport in Germany received 56,330 noise-related complaints, a 30% increase over the same month in 2001. The same year, residents living near a rural airport outside London, England, were submitting 100 petitions daily, objecting to proposals for three new runways at the site.

In March 2003, representatives from eight neighborhoods in Portland, Oregon, showed up for a city council hearing convened to discuss dozens of expansion projects for Portland International Airport. The airport was already a busy one: in 2002 it handled 12.2 million passengers and about 29,000 containers of air cargo. “The impacts are tremendous on the neighborhoods under the flight paths,” testified one neighborhood representative, Jean Ridings. “People move in and move [right back] out. It’s becoming a disaster.” In response, the airport has initiated a multiyear, multimillion-dollar effort to study the sound impact of the airport, which locals hope will lead to a plan to reduce airport noise.

Noise Free America is seeking to file a class-action lawsuit against the makers of boom car equipment. Ted Rueter, Noise Free America’s director and an assistant professor of political science at DePauw University in Greencastle, Indiana, says one group member has written a legal brief on the topic and has approached several public-interest law firms seeking representation, with no takers so far. Rueter says Noise Free America will continue to pursue the suit.

A lot of money is being made from disturbing the peace, charges Mark Huber, communications director for Noise Free America. “By using paid lobbyists in Washington, D.C., and in state legislatures, the automobile and entertainment industries are quietly removing obstacles protecting the public against noise,” Huber says. “Try to get a noise control law passed through a state legislature and see what happens. We tried to get a boom car law enacted in the Virginia General Legislature, but right here in Richmond there are at least fifty car clubs, all of which are politically active. So our legislation disappeared.”

Stephen McDonald, vice president of government affairs for the Washington, D.C.–based Specialty Equipment Market Association (SEMA), denies that any powerful lobby exists and is working against the best interests of society. SEMA represents manufacturers, distributors, retailers, and installers of specialty automotive equipment, including boom car equipment. “Our prime focus is representing the interests of businesses that sell exhaust systems,” McDonald says. “But that doesn’t mean we want the products to increase noise to a level where it becomes objectionable. We do need to strike a balance, though, between what is acceptable for a neighborhood and what’s fair to people who want to customize their cars.”

Anti-noise activists say that Europe and several countries in Asia are more advanced than the United States in terms of combating noise. “Population pressure has prompted Europe to move more quickly on the noise issue than the United States has,” Hume says. In the European Union, countries with cities of at least 250,000 people are creating noise maps of those cities to help leaders determine noise pollution policies. Paris has already prepared its first noise maps. The map data, which must be finished by 2007, will be fed into computer models that will help test the sound impact of street designs or new buildings before construction begins.

In the United States, the Noise Control Act of 1972 empowered the EPA to determine noise limits to protect the public health and welfare, and to establish a noise control office. Congress did establish the Office of Noise Abatement and Control (ONAC), as well as federal standards for business, industries, and communities, and it did begin researching the effects of sound exposures. In 1982, however, the Reagan administration defunded the office. “We are no longer doing research on noise,” says Kenneth Feith, an EPA senior scientist and policy advisor. “We just don’t have the money or staff to do it.”

Activists believe that closing the ONAC has had a tremendous negative effect at the state and local level. “The U.S. has long since given up its lead in regulating noise, and because of that there has been no consistency in implementing local noise regulations,” Huber says. The Noise Control Act, though still on the books, is essentially toothless.

In the mid-1990s, people in the borough of Queens, New York, who lived under the flight paths of La Guardia Airport, took their concerns about noise to Representative Nina Lowey (D–NY). “I could see that noise is a serious public health issue, and so I decided to do something about it,” Lowey says. In 1997 the congresswoman introduced legislation that’s become known as the Quiet Communities Act (HR 536), which provided for the refunding of the ONAC and for $21 million to be spent annually on noise reduction. Among other measures, the money would be used to carry out a national noise assessment program to identify trends in noise exposure and response, develop and disseminate information and public education materials on the health effects of noise, and establish regional technical assistance centers, which would use the resources of universities and private organizations to assist state and local noise control programs.

“More and more communities are being affected by airports, trains, and railways,” Lowey says. “We need a national office to coordinate policy. That’s common sense to me. The federal government has to play a larger role on the noise issue. Otherwise, we will continue to lag behind other parts of the world in combating noise.” While Lowey remains optimistic that the legislation will eventually pass, other sources doubt that it will happen, noting that the proposed legislation has been introduced and rejected several times.

Activists in other countries say they too want the United States to play a more leading role on the noise issue. “Re-establishing the ONAC would be a huge move in the right direction,” says Hans Schmid, the Vancouver, Canada–based president of the Right to Quiet Society. “That will show that the United States is serious about the noise issue. If the United States leads, other countries, especially Canada, will follow.”

But as in other areas of environmental health, merely having a more powerful government agency in place that can set more regulations is not the ultimate answer, according to other experts. Regulations provide an important foundation, Stephenson says, but better education of workers, consumers, businesses, and citizens is critical. “We’ve found that in some factories as many as one-third of the workers who have significant hearing loss don’t wear hearing protectors, even though the factory has a comprehensive hearing conservation program in place,” he says.

Bronzaft stresses that governments worldwide need to increase funding for noise research and do a better job coordinating their noise pollution efforts so they can establish health and environmental policies based on solid scientific research. “Governments have a responsibility to protect their citizens by curbing noise pollution,” she says.

Feith agrees. “The EPA had a successful educational program in the nineteen seventies in which we went to schools and educated students about noise,” he says. “When students took the message home, they helped increase the sensitivity to the noise issue. We need more programs like that to educate the public about noise.”

In the meantime, some facilities are doing what they can to help themselves to a quieter environment. Although peace and quiet are essential prerequisites for a healing environment, a Mayo Clinic study published in the February 2004 issue of the *American Journal of Nursing* showed that peak noise levels during the clinic’s morning shift change rivaled the excruciating sound of a jackhammer. The study further showed that a few simple changes—for example, holding staff reports at shift change in an enclosed room (rather than at the nurses’ station) and replacing roll-type paper towel dispensers with quieter models—reduced peak noise levels at shift change by 80%.

Similarly, the din of overhead pagers, which can reach 80 dBA, inspired the developers of the Woodwinds Health Campus in Woodbury, Minnesota, to build the facility with a staff location sensor and badge system, among other sound-friendly features. Staff can be located in just about any area of the Woodwinds campus without being paged. “We have developed an innovative approach to reducing noise in our hospital while fostering a healing environment,” says Cindy Bultena, executive lead of healing and clinical coordination for Woodwinds. “Our change sounds simple enough, but it’s a very radical one for hospitals.”

By delivering their patients and staff from decibel hell, facilities like Woodwinds and the Mayo Clinic have scored one small victory in the ongoing battle against noise pollution. Their initiative, moreover, shows that given the pervasiveness and harmful effects of noise, governments, communities, and organizations worldwide will need to be creative and aggressive in addressing what will certainly continue to be one of the 21st century’s most important environmental health issues.

## Figures and Tables

**Figure:**
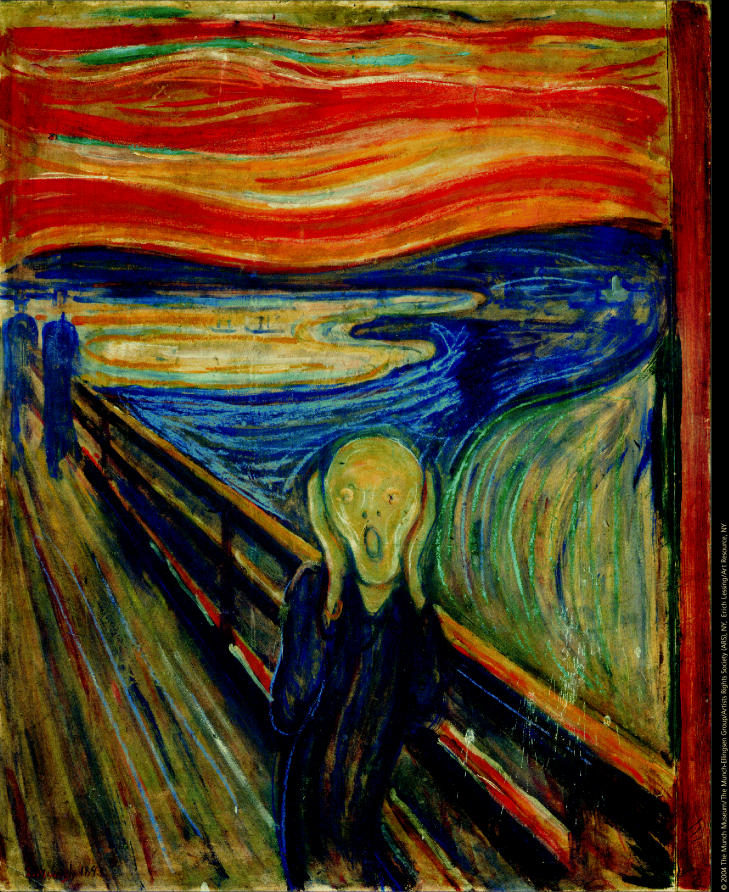


**Figure:**
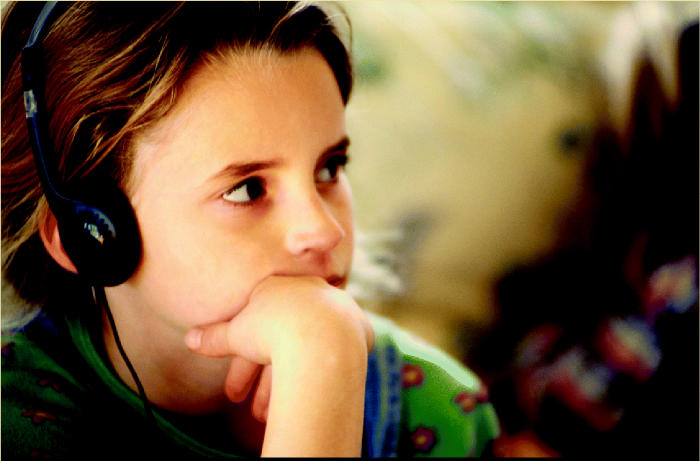
**On the increase.** Our technological society encourages the propagation of noisy devices, and children are being exposed earlier than ever to an abundance of electronic noise.

**Figure:**
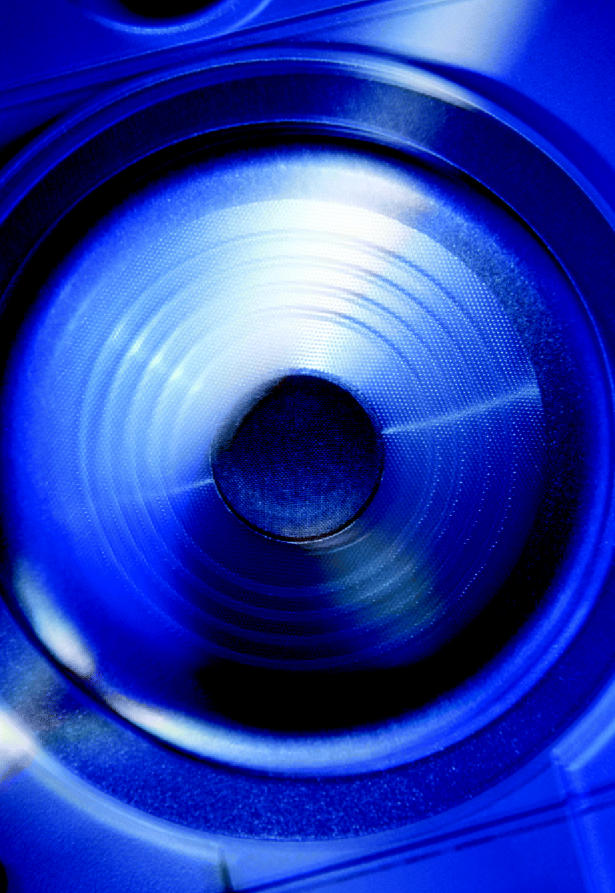
**On the street.** Booming bass is quickly becoming the sound-track of urban life.

**Figure:**
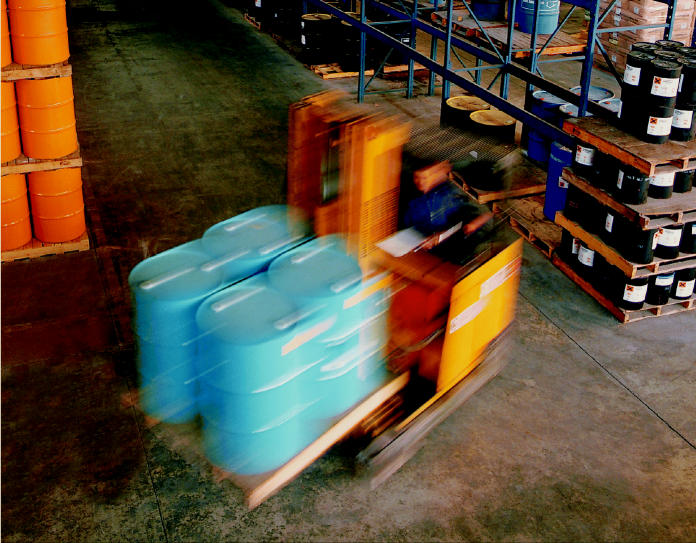
**On the job.** Occupational noise is pervasive throughout many industries and may cause serious damage despite regulations to protect workers’ hearing.

**Figure:**
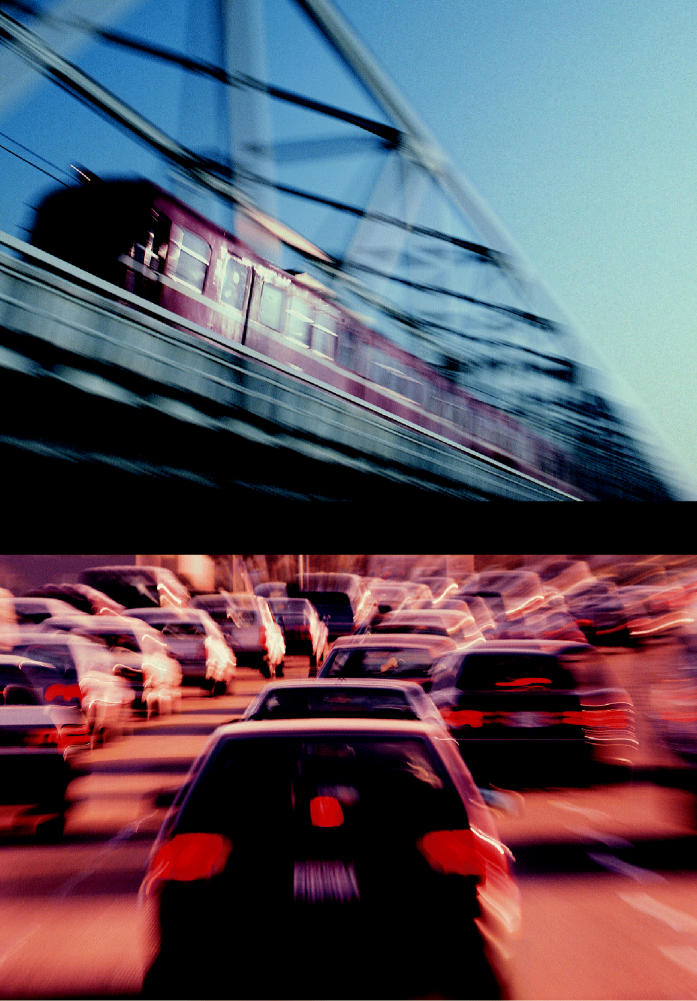
**On the go.** Transportation sound is perhaps the largest contributor to urban noise pollution.

**Figure:**
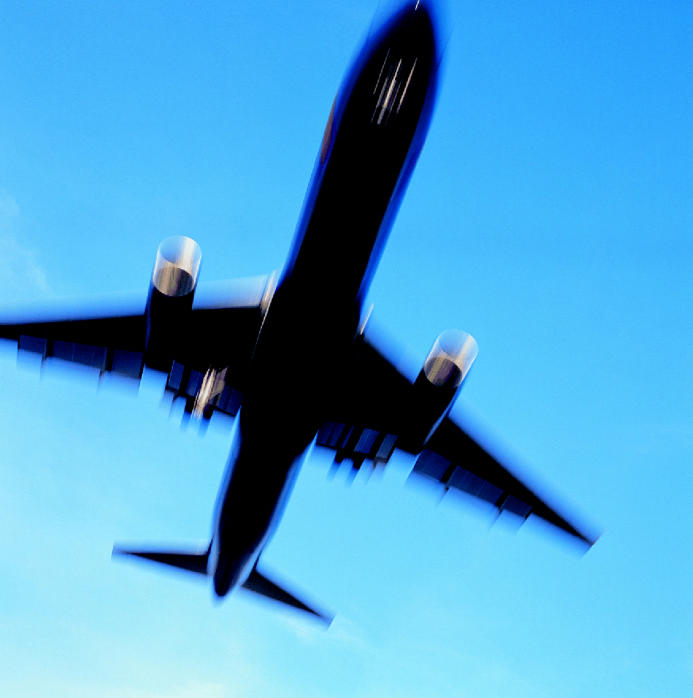
**On the way up.** Problems from airplane and airport noise are increasing as more and more flights take off over residential areas.

**Figure:**
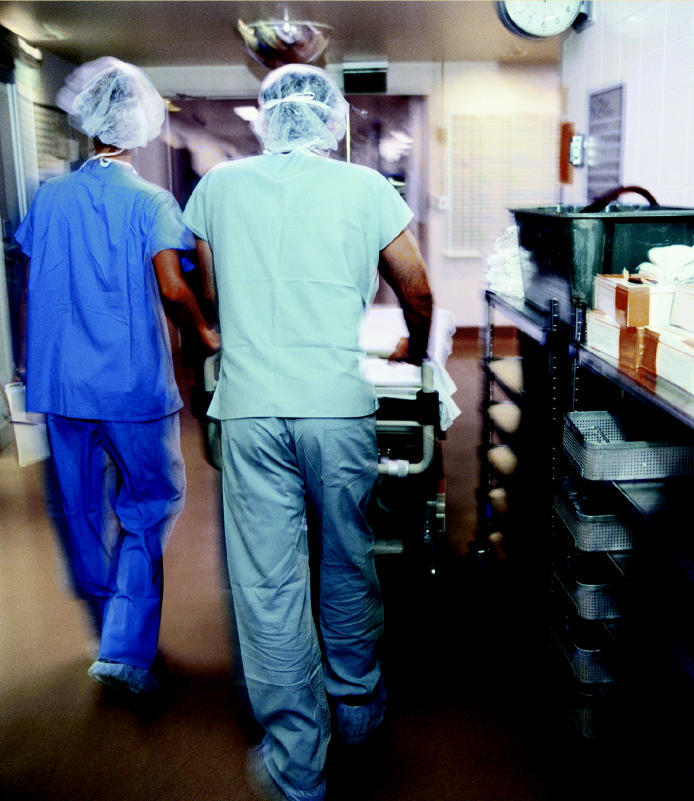
**On the mend?** Hospitals can be some of the noisiest public locations, but some health care facilities are actively fighting noise in the interest of better patient care.

**Table:** Counting Decibels

**Device/Situation**	dBA*
Grand Canyon at night, no birds, no wind	10
Quiet room	28–33
Computer	37–45
Floor fan	38–70
Refrigerator	40–43
Normal conversation	40
Forced-air heating system	42–52
Radio playing in background	45–50
Clothes washer	47–78
Dishwasher	54–85
Bathroom exhaust fan	54–55
Microwave oven	55–59
Normal conversation	55–65
Laser printer	58–65
Hair dryer	59–90
Window fan on “high” setting	60–66
Alarm clock	60–80
Vacuum cleaner	62–85
Push reel mower	63–72
Sewing machine	64–74
Telephone	66–75
Food disposal	67–93
Inside car with windows closed, traveling at 30 miles per hour	68–73
Handheld electronic game	68–76
Inside car with windows open, traveling at 30 miles per hour	72–76
Electric shaver	75
Air popcorn popper	78–85
Electric lawn edger	81
Electric can opener	81–83
Gasoline-powered push lawn mower	87–92
Average motorcycle	90
Air compressor	90–93
Weed trimmer	94–96
Leaf blower	95–105
Circular saw	100–104
Maximum output of stereo	100–120
Chain saw	110
Average snowmobile	120
Average fire crackers	140
Average rock concert	140

* Measurements are approximate and may vary by source.

**Sources:** National Institute on Deafness and Other Communication Disorders, Environmental Protection Agency, Noise Pollution Clearinghouse.

